# Hegemonic medicine and self-managed abortion: reclaiming Latin American feminists’ contributions to knowledge and practice development

**DOI:** 10.1080/26410397.2025.2576263

**Published:** 2025-10-17

**Authors:** Sara Larrea, Suzanne Veldhuis

**Affiliations:** a Independent Consultant, Alicante, Spain; b PhD Candidate, El Colegio de la Frontera Sur, San Cristóbal de Las Casas, Mexico.

**Keywords:** autonomy, biomedicine, demedicalisation, medication abortion, person-centredness, self-managed abortion

## Abstract

In this commentary, we challenge the narrative that presents self-managed medication abortion (SMA) as a recent "discovery" of Western biomedicine. Drawing on our lived experience and a literature review, we compare how different key concepts – autonomy, empowerment, person-centred care, privacy, confidentiality, and demedicalisation – are understood by the feminist SMA movement in Latin America, versus hegemonic medicine. We argue that when the radical ideas of the feminist SMA movement are appropriated by mainstream health systems without proper recognition of their political foundations, they lose their original meaning and may fail to achieve the positive outcomes envisioned by activists. For instance, autonomy, privacy, and demedicalisation may result in individuals feeling unsupported, while person-centred care often means merely being treated without stigma or violence. Maintaining medical control over SMA restricts access, while neoliberal policies that promote “self-care” and the commodification of abortion pills enable states to evade responsibility with regard to abortion care. We advocate for integrating key concepts taken from SMA activism into formal health systems, while honouring their origins and political significance. This requires meaningful, horizontal collaborations between SMA activists and biomedical professionals, acknowledging that SMA activists are abortion experts and should be treated as such.

## Résumé

Dans ce commentaire, nous remettons en question le discours qui présente l’avortement médicamenteux autogéré comme une récente « découverte » de la biomédecine occidentale. Nous fondant sur notre expérience vécue et sur un examen des publications, nous comparons la manière dont différents concepts clés – autonomie, autonomisation, soins centrés sur la personne, respect de la vie privée, confidentialité et démédicalisation – sont compris par le mouvement féministe autour de l’avortement médicamenteux autogéré en Amérique latine, par rapport à la médecine hégémonique. Nous soutenons que, lorsque les idées radicales du mouvement féministe autour de l’avortement médicamenteux autogéré sont reprises par les systèmes de santé dominants sans que leurs fondements politiques soient dûment reconnus, elles perdent leur sens originel et risquent de ne pas atteindre les résultats positifs escomptés par les militants. Par exemple, l’autonomie, le respect de la vie privée et la démédicalisation peuvent conduire les personnes à ressentir un manque de soutien, alors que les soins centrés sur la personne se résument souvent à un traitement sans stigmatisation ni violence. Maintenir le contrôle médical sur l’avortement médicamenteux autogéré restreint l’accès, tandis que les politiques néolibérales qui encouragent les « soins autogérés » et la marchandisation des pilules abortives permettent aux États de se soustraire à leurs responsabilités en matière d’avortement. Nous préconisons l’intégration des principaux concepts issus du militantisme autour de l’avortement médicamenteux autogéré dans les systèmes de santé formels, tout en respectant leurs origines et leur importance politique. Cela nécessite une véritable collaboration horizontale entre les militants en faveur de l’avortement médicamenteux autogéré et les professionnels biomédicaux, reconnaissant que ces militants sont des experts en matière d’avortement et doivent être traités comme tels.

## Resumen

En este comentario, cuestionamos la narrativa que presenta la autogestión del aborto (AGA) con medicamentos como un "descubrimiento" reciente de la biomedicina occidental. A raíz de nuestras vivencias y una revisión bibliográfica, comparamos cómo diferentes conceptos clave, tales como autonomía, empoderamiento, atención centrada en la persona, privacidad, confidencialidad y desmedicalización, son entendidos por el movimiento feminista de AGA en América Latina, en comparación con la medicina hegemónica. Argumentamos que, cuando las ideas radicales del movimiento feminista de AGA son apropiadas por los sistemas de salud convencionales sin el debido reconocimiento de sus fundamentos políticos, pierden su significado inicial y posiblemente no logren los resultados positivos previstos por activistas. Por ejemplo, la autonomía, la privacidad y la desmedicalización podrían causar que la persona no se sienta apoyada, mientras que la atención centrada en la persona a menudo significa simplemente recibir atención sin estigma o violencia. Mantener el control médico sobre la AGA limita el acceso, mientras que las políticas neoliberales que promueven “el autocuidado” y la mercantilización de las pastillas abortivas permiten a los estados evadir su responsabilidad relacionada con los servicios de aborto. Abogamos por integrar en los sistemas de salud formales los conceptos clave adoptados del activismo por la AGA, y a la vez honrar sus orígenes y significado político. Para ello se necesitan colaboraciones horizontales significativas entre activistas de AGA y profesionales biomédicos, y reconocer que los activistas de AGA son expertos en aborto y deben ser tratados como tal.

## Introduction

As longtime abortion researchers and activists working both in and from Latin America and the Caribbean,* we have extensive experience witnessing how medication abortion and its self-management variants are misrepresented as recent “discoveries” of the Western medical establishment. Studies on self-managed medication abortion (SMA) often fail to mention that this method was successfully used outside of clinical settings long before being studied within formal medical systems.^1,2^ By omitting the origins of the practice, these studies implicitly credit a handful of Western physicians and researchers with a “discovery” that was actually made by women and pregnant individuals themselves. The narrative that there is “no precedent” to the knowledge created by clinical studies reflects a common colonialist narrative in biomedicine.^3,4^

In reality, the notion that abortion could be safely self-managed without medical supervision originates from the practice, first documented in Brazil in the 1980s, of women using misoprostol off-label to induce abortions.^5^ While clinical research had an important role in developing scientific knowledge that made medication abortion acceptable to the medical establishment and policymakers, it was feminist movements, particularly in Latin America, that popularised medication abortion and created the grassroots infrastructures that made it accessible to abortion seekers. Feminist collectives supporting SMA have also contributed to building scientific knowledge about their innovative practices.^6–8^

Despite the vast scientific and practical evidence on its safety, the medical establishment often described SMA as a desperate solution for settings where safe abortion methods are unavailable.^5,9^ However, recent developments – such as the digitalisation of medical systems, the unprecedented interest in “self-care” and remote models fostered by the COVID-19 pandemic and heightened legal restrictions on abortion in the United States – have increased scientific and medical communities’ interest in SMA.^2,10–12^ In this new era for medication abortion, the lack of acknowledgment of the Latin American SMA movement continues to be the norm.

In this commentary, we argue that the lack of recognition of the contributions of feminist activists to this development is closely tied to the dominant role of biomedicine. We also posit that when the radical ideas and insights from the feminist SMA movement are appropriated by mainstream health systems, they lose their original meaning and fail to serve their primary goal: democratising abortion access and fostering conditions for enhanced bodily autonomy and reproductive justice.

## SMA and feminist models of care

Self-managed medication abortion (SMA) includes practices such as self-assessing eligibility and abortion success, self-sourcing, and self-administering the pills.^11^ SMA exists along a spectrum, ranging from more medicalised models – for example, where pregnant people take the pills at home, but physicians supervise parts of the process either in person or through telemedicine – to demedicalised, autonomous models, where individuals manage the entire process themselves or with support from feminist activists.^13^

For over two decades, feminists in Latin America have supported abortion access in various ways – from hotlines that provide information on how to obtain and safely use abortion pills, to *acompañamiento* providing practical, technical, and emotional support for people self-managing their abortions or accessing clinical abortion care, through one-on-one or group support sessions, in-person or virtual.^6–8,14,15^ By working also in (scientific) knowledge creation, they have extended SMA support beyond the gestational limits set by laws and protocols,^6,7^ developed pain management strategies that incorporate alternative medicine,^7^ and created communication materials to address the unique needs of migrants and people with disabilities.^16^

Most *acompañantes* are part of political collectives or networks whose work extends beyond abortion access. While they are diverse, feminist SMA models share core characteristics such as centring autonomy, establishing non-hierarchical relationships with the people they support, sharing scientific information in accessible formats, and making sure that the person having the abortion leads the process and makes all relevant decisions.

## Biomedicine and the hegemonic medical model

Biomedicine – the branch of Western medicine based on the idea that illness is the product of deviations from a universal biological standard^17^ – is currently the predominant form of healthcare around the world (other forms are, for example, the traditional medicine of indigenous American and Asian cultures). Globally, social and cultural ideas around health and healthcare have been permeated by biomedical thinking, establishing it as the hegemonic medical model (HMM).

Medical anthropologists characterise the HMM as biologistic, individualistic, medicalised, and pragmatic, highlighting the asymmetric patient-doctor relationships and the commodification of health in hegemonic medicine.^4^ Feminist and anti-racist scholars have added that biomedicine is androcentric, heteronormative, classist, and colonialist: it considers the white, heterosexual, middle-class male body as the “universal standard”, while treating other bodies as deviations.^3,18–20^ In this regard, biomedicine also plays a role in social control.^21^

Since its origins in the eighteenth century, hegemonic medicine has delegitimised any health practices not grounded in scientific positivism and rationality, subordinating, ignoring, questioning, stigmatising, banning, or appropriating alternative models, including those based on self-care and community practices, such as SMA.^4,21,22^ Examples of how the HMM has incorporated SMA practices without considering the feminist principles in which they are rooted include the implementation of telemedicine services during the COVID pandemic^2,12^ and the incorporation of the “innovative” practice of supporting SMA by international organisations.^23,24^

In the following sections, we compare how key concepts related to SMA are understood by the feminist SMA movement in Latin America and by the hegemonic medical narrative.

## Autonomy and empowerment

In the ‘90s, when SMA emerged as an alternative in settings where clinical abortion services were either inaccessible or non-existent, grassroots feminist activists quickly recognised its empowering potential. They positioned a specific SMA practice, *aborto autónomo*, as a political statement that rejects the power and control exerted by nation-states and their institutions – including health institutions – over gendered bodies, firmly grounding the practice in a human rights-based framework^25^ and building relational notions of autonomy and freedom.^26^ At the same time, feminist activists hold governments and health institutions accountable, demanding high-quality abortion care for those who seek or need medical assistance,^6^ a stance shared by the World Health Organization (WHO).^11^

Feminist support for SMA is rooted in deep trust and respect for bodily autonomy, and enables transformative, empowering, and peer-supported abortion experiences.^6,7^ In feminist models of care, the decision to have an abortion is never questioned. While activists provide information and support, the process is led entirely by the person having the abortion. Those who self-manage their abortions value the autonomy it facilitates.^27,28^ Positive SMA experiences are often linked to feminist support and accompaniment: non-judgemental, respectful, and kind care, provided promptly.^29,30^ On the other hand, when abortion pills are used without support, the experience can be distressing.^5,31^

In contrast, the HMM departs from the premise that people are not able to make the right choices about their own bodies and health. This is what Menéndez coins as “the ignorant patient”.^4^ Under this framework, medical supervision is needed to ensure appropriate care and minimise risks.^32^ *Allowing* self-care is only seen as safe under very specific conditions, which must be previously validated by the medical establishment.^33^ This is exemplified by the perceived need for scientific evidence to show, for example, that “*women can follow directions* and safely take the mifepristone out of the office”.^34^

Health systems reinforce the commodification of health in the HMM by adopting SMA practices, as it allows the system to avoid assuming responsibility for ensuring (but not imposing!) high-quality abortion services. The integration of SMA elements into institutional medical systems (via telemedicine or other models) may also align with neoliberal reforms, as well as with governments and health providers unwilling to facilitate abortion access. SMA practices without appropriate support allow reducing the size, resources, and costs of the formal health system,^33^ and enable physicians to distance themselves from abortion, and avoid “practicing” it directly.^35,36^

From a Latin American feminist perspective, promoting self-care and autonomy should not come at the expense of the existence of high-quality abortion services, with trained and empathic personnel, accessible to all who want or need facility-based care. Self-care and autonomy should be grounded in collective care, and not reduced to the neoliberal notion of individual responsibility, which allows states and health institutions to shirk their obligations.^37^

## Centred on people's needs, desires, and possibilities

Feminist SMA initiatives base their practices on the individual needs, desires, and possibilities of each person they accompany. Rooted in their in-depth knowledge of the local settings, they tailor support to the social, economic, and emotional conditions, as well as the ideas and knowledge of abortion seekers. This explains the diversity of models across Latin America and globally.

In practice, this means that the terms and duration of the contact and follow-up with the *acompañante,* and medical aspects such as the method, ultrasounds, number of misoprostol doses, and ways of confirming abortion success, are offered as options and finally determined by the aborting person.^15^ These are examples of how activists systematically centre autonomy, create non-hierarchical relationships, and practice active listening, which are fundamental changes in abortion care that push the limits of biomedicine. Some authors have described this as person-centred care.^29^

However, feminist SMA practices are substantially different from what hegemonic medicine understands as person-centredness. In the global context of abortion stigma and criminalisation, activists and specialised researchers have strategically used this concept to tackle the violence and mistreatment abortion seekers often face within mainstream medical care.^38,39^ As a result, researchers and practitioners currently consider person-centredness an important dimension of high-quality abortion care, defined as a respectful and responsive approach to individuals’ preferences, needs, expectations, and values. It also includes comprehensive counselling, non-judgemental, non-discriminatory, and supportive care.^38,40^ However, when measuring or implementing “person-centred” care in clinical abortion services, practitioners often reduce these definitions to indicators such as introducing themselves, calling patients by their name, having respectful manners, showing they care, maintaining privacy and confidentiality, and asking for consent before procedures, etc.^11,38^ From a feminist perspective, these elements are basic human interactions, and their inclusion in the context of medical care is expected as part of normal, non-discriminatory, and stigma-free care. Moreover, the one-size-fits-all principle of biomedicine often falls short of the flexibility required to truly adapt the provision of care to individual needs.

## Privacy and confidentiality

The possibility of keeping abortions private and confidential – choosing with whom to share the information about the decision to abort – has been one of the arguments of feminist activists to advocate for SMA. As most cases of abortion criminalisation start with reports from health professionals,^41^ feminist activists deeply value the political potential of being able to abort without the involvement of health workers. At the same time, feminist activism advocates for the decriminalisation and destigmatisation of abortion, insisting that it should be recognised as part of normal human life that need not be hidden.

However, the HMM's narrow focus on maintaining privacy and confidentiality without the feminist political demands for decriminalisation and normalisation can reinforce abortion stigma and exceptionalism by keeping abortion in the private sphere. It also strengthens the notion that abortions require special treatment in comparison to other medical procedures.

Finally, the liberal argument for medication abortion to be performed privately without medical supervision carries the risk of absolving states, medical institutions, and societies of their responsibility, and so does approving autonomy without offering support and alternatives when needed or desired. Likewise, reducing the issue to an individual’s private matter may pave the way to commodification. Latin American feminists have historically resisted attempts to commodify healthcare, dismantle healthcare infrastructures, and privatise health systems, promoted by the structural adjustment policies of multilateral financial organisations and neoliberal governments.^42^ The feminist demand for recognition and support of SMA as a safe and viable alternative should be understood in this context. Latin American feminist activisms insist that, while SMA needs to be recognised as a valid option, it should not replace the need for strong healthcare systems that ensure equal access to quality care.

## Demedicalisation

Demedicalisation is a core concept in SMA models of care.^43,44^ While medicalisation refers to the definition of social issues in medical terms, thereby placing them under medical control,^45^ the feminist struggle for the demedicalisation of abortion implies rejecting this control over women and other pregnant people’s bodies, while advocating for a broad range of options and supporting strong medical systems that ensure access to care for individuals with diverse needs and desires.

Since the discovery of abortion pills, significant steps towards safe abortion care have been taken place within formal health systems. For instance, the WHO now recommends SMA for pregnancies up to 12 weeks and no longer considers ultrasounds mandatory. In addition, health systems largely acknowledge that no-touch protocols, telemedicine, and task-shifting procedures are safe, effective, and acceptable.^11^ They recognise these examples of demedicalisation as strategies to increase accessibility and reduce costs, but these “innovative” models of care do not always increase autonomy or reduce medical control. In most cases, abortion care remains under the supervision of health professionals and is strictly regulated by medical protocols.

This highlights, again, the fundamental difference between the SMA movement and biomedicine. For feminist activists, demedicalisation is a strategy to regain control and bodily autonomy, grounded in the belief that abortion is a natural process that should be part of community care.^46^ Health services are resources that need to be accessible when needed, rather than a requirement for ensuring safety. However, for hegemonic medicine, abortion remains inherently dangerous and requires medical supervision to guarantee its safety. Health systems and professionals have *granted* demedicalisation and autonomy only after extensive research on the safety of every single one of its aspects, without questioning power inequalities between the “provider” and the “patient”.

If health systems limit demedicalisation to allowing the sale of abortion pills at pharmacies and allowing home abortions, this could maintain the barriers for disenfranchised populations who may struggle to access information, support, and the pills themselves.^47^

## How can hegemonic medicine integrate the learnings of Latin American feminist SMA?

The first logical answer is that it can't: biomedicine and Latin American feminisms’ views of abortion and care are fundamentally different. When SMA practices are incorporated into medical systems without the feminist political foundations that drive them, they may not always produce the positive outcomes advocated by activists, as they remain under medical control and are bound by medical protocols. Autonomy, privacy, and demedicalisation may translate into individuals feeling abandoned, lacking support, while person-centred care means – at most – being treated without stigma and violence. Meanwhile, regulations hinder access by determining the extent to which self-management is allowed and when it can be applied. On the other hand, neoliberal politics may promote “self-care” and the commodification of abortion pills as a way for states to avoid taking responsibility for guaranteeing access to abortion care for all who need it.

Nonetheless, our experience as feminist health professionals working on abortion access in Latin America has shown us that meaningful collaborations between feminisms and biomedicine are possible and can be fruitful.^16,48–50^ *Acompañantes* in Argentina and Mexico are currently training medical professionals on medication abortion provision, especially in advanced pregnancies. Also in Argentina, feminist organisations participated in the design of the public policy implemented after abortion legalisation in 2020, while in Colombia, activists and medical professionals united in *Causa Justa,* which led to the decriminalisation of abortion up to 24 weeks.^44,48–50^ These examples show that genuine collaboration is possible under specific conditions, such as trust and mutual recognition.

SMA activism advocates for a fundamental change in the power dynamics within health systems. It challenges the view that women's and gender non-conforming individuals’ knowledge of their own health is less valid than medical knowledge. These tensions are not unique to Latin America. Therefore, this shift has the potential to transform not only how abortion care is approached, but also how other health issues – for example, menstruation and contraception – are understood and treated, by creating new and flexible models of care that can be implemented globally.

We call for the integration of central concepts to SMA activism – such as autonomy, empowerment, person-centred care, privacy, confidentiality, and demedicalisation – into formal health systems, while honouring their origins and maintaining their political significance. Achieving this requires meaningful, horizontal collaborations between SMA activists and biomedical professionals, based on the acknowledgment that SMA activists are abortion experts and should be treated as such.^46^ In practice, this requires decriminalising abortion, including its self-management; creating spaces for meaningful participation by activists in the development of protocols and models of care within the formal health system; and securing sufficient public funding and support to ensure that all options – i.e. clinical care, medical support for self-management, and autonomous abortion – are available for those who need them.

## Footnotes

* S. Larrea is an Ecuadorian sociologist and public health professional who has worked on abortion access and research since 2006. S. Veldhuis is a Dutch medical doctor who has worked as an abortion provider, activist, and researcher in Chiapas, Mexico, since 2012. Both authors are cis-women and have been members of feminist organisations working on abortion access, both in Latin America and the Caribbean, and globally.
